# Genetic variants in *ALDH1B1* and alcohol dependence risk in a British and Irish population: A bioinformatic and genetic study

**DOI:** 10.1371/journal.pone.0177009

**Published:** 2017-06-08

**Authors:** Michael J. Way, M. Adam Ali, Andrew McQuillin, Marsha Y. Morgan

**Affiliations:** 1 Molecular Psychiatry Laboratory, Division of Psychiatry, University College London, London, United Kingdom; 2 UCL Institute for Liver & Digestive Health, Department of Medicine, Royal Free Campus, University College London, London, United Kingdom; Kunming Institute of Zoology, Chinese Academy of Sciences, CHINA

## Abstract

Alcohol is metabolized in the liver *via* the enzymes alcohol dehydrogenase (ADH) and aldehyde dehydrogenase (ALDH). Polymorphisms in the genes encoding these enzymes, which are common in East Asian populations, can alter enzyme kinetics and hence the risk of alcohol dependence and its sequelae. One of the most important genetic variants, in this regards, is the single nucleotide polymorphism (SNP) rs671 in *ALDH2*, the gene encoding the primary acetaldehyde metabolizing enzyme ALDH2. However, the protective allele of rs671 is absent in most Europeans although *ALDH1B1*, which shares significant sequence homology with *ALDH2*, contains several, potentially functional, missense SNPs that do occur in European populations. The aims of this study were: (i) to use bioinformatic techniques to characterize the possible effects of selected variants in *ALDH1B1* on protein structure and function; and, (ii) to genotype three missense and one stop-gain, protein-altering, non-synonymous SNPs in 1478 alcohol dependent cases and 1254 controls of matched British and Irish ancestry. No significant allelic associations were observed between the three missense SNPs and alcohol dependence risk. The minor allele frequency of rs142427338 (Gln378Ter) was higher in alcohol dependent cases than in controls (allelic *P* = 0.19, OR = 2.98, [0.62–14.37]) but as this SNP is very rare the study was likely underpowered to detect an association with alcohol dependence risk. This potential association will needs to be further evaluated in other large, independent European populations.

## Introduction

Alcohol is metabolized in the liver to acetaldehyde and acetate *via* the enzymes alcohol dehydrogenase (ADH) and aldehyde dehydrogenase (ALDH). Functional polymorphisms in the genes encoding ADH and ALDH are associated with alterations in enzyme kinetics [1, 2], which, in turn, may determine the rates of production and removal of the toxic intermediate metabolite acetaldehyde. High circulating acetaldehyde concentrations are associated with a number of unpleasant systemic effects, including: facial flushing, tachycardia, nausea, headache and even collapse and death [3, 4]. In consequence, individuals carrying certain functional *ADH* and *ALDH* variants tend to limit their alcohol consumption and so are, in effect, protected from developing alcohol dependence and the medical consequences of harmful drinking [5–8].

There are significant differences in the frequencies of these functional *ADH* and *ALDH* variants between ethnic groups. The prevalence of these variants is higher in the Far East and very much lower in Europe. Thus, for example, the missense single nucleotide polymorphism (SNP) rs1229984 (Arg48His) in *ADH1B*, which increases the maximal velocity at which alcohol is oxidized to acetaldehyde by over 100-fold [3, 9], is found in 19 to 91% of East-Asians and 10 to 70% of West-Asians [7], but at rates ranging from zero to 10% in other populations [10,11]. Nevertheless, there is consistent evidence that the protection against the development of alcohol dependence conferred by carriage of this variant extends to these populations as well [7,12–15].

Similarly, a missense SNP, rs671 (Glu504Lys) in *ALDH2*, which inactivates the mitochondrial isoform of ALDH2 causing a loss of up to 90% of its enzymatic activity and 20-fold elevations in circulating acetaldehyde levels following alcohol consumption, is found in 30 to 50% of East-Asians [16] but is absent in Europeans [17]. However, other genes, for example *ALDH1B1*, have the capacity to oxidize acetaldehyde at physiologically relevant rates [18] and may be of importance in relation to alcohol dependence in European populations. *ALDH1B1* is located on chromosome 9 and its encoded protein, ALDH1B1, has 75% amino acid sequence homology with ALDH2. In addition, in common with ALDH2, it is expressed in liver mitochondria and is predicted to form homo-tetramers comprised of four ALDH1B1 monomer subunits [18]. Computational analysis predicts that ALDH1B1 monomer subunits preferentially hetero-tetramerize with ALDH2 rather than forming homo-tetramers amongst themselves; this provides a hypothetical explanation for why inactivation of ALDH2 in carriers of the *ALDH2* 504Lys allele is not compensated for by ALDH1B1 [19].

A number of missense SNPs have been identified in *ALDH1B1*, which are thought likely to alter the structure of its protein product the enzyme ALDH1B1 [20–22]. Jackson et al [19], undertook computational modelling of human recombinant ALDH1B1 and investigated the functional effects of three non-synonymous SNPs in *ALDH1B1 viz*: rs2073478 (Arg107Leu), rs2228093 (Ala86Val) and rs4878199 (Val253Met) *in vitro*. They showed that presence of the Val86 allele of rs2228093 abolished enzyme activity whereas the other two SNPs appeared to have no effect. The association between two of these SNPs, rs2073478 (Arg107Leu) and rs2228093 (Ala86Val) and alcohol consumption levels and the risk for developing alcohol dependence has been explored but with no robust or replicated findings [20,23–25]. Thus, there is some functional evidence suggesting that non-synonymous SNPs in *ALDH1B1* might affect enzyme activity and hence alcohol metabolism. There is also limited and conflicting genetic evidence for their role in modifying the risk for developing alcohol dependence in man.

The primary aims of this study were: (i) to use bioinformatic techniques to identify and characterize non-synonymous SNPs in *ALDH1B1*; and, (ii) to investigate, using a case control design, whether these functional variants associate with alcohol dependence risk in a large, carefully characterized population of British and Irish ancestry.

## Materials and methods

### Bioinformatic analysis

#### Selecting variants in *ALDH1B1*

A search for nonsense variants with any notated frequency, and missense variants with minor allele frequencies (MAF) exceeding 5% in populations of European ancestry was undertaken in the 1000 Genomes Project build 37 phase I integrated data in the relevant region (chr9: 38,395,746–38,397,299) [21].

Four, non-synonymous variants fulfilled the selection criteria: three were missense variants; rs2073478 (Arg107Leu), rs2228093 (Ala86Val) and rs4878199 (Val253Met); and one stop/gain variant, rs142427338 (Gln378Ter). These variants are common in the reference 1000 genomes European ancestry sub-populations [21]. The allele frequencies for rs2073478 (Arg107Leu) and rs2228093 (Ala86Val) are largely similar in the British, Toscani and Iberian groups, with the frequency distributions in the Finnish group varying most noticeably from the rest (S1 Fig).

#### Missense variant functionality prediction

The likelihood that the three missense variants were damaging was investigated using the functional prediction software PolyPhen-2 [26]; rs2073478 (Arg107Leu) and rs2228093 (Ala86Val) were predicted to be damaging while the mutation in the third, rs4878199 (Val253Met) was predicted to be benign (Table 1). This software cannot be used to predict the potential damaging effects of loss of function variants, such as rs142427338 (Gln378Ter), as the rare allele of this SNP encodes a stop codon.

**Table 1:** PolyPhen-2* functional prediction analysis of the three *ALDH1B1* missense variants.

SNP identity	Amino acid change	PolyPhen-2 score (0–1)	PolyPhen-2 prediction
rs2073478	Arg107Leu	0.46	Possibly damaging
rs2228093	Ala86Val	0.99	Probably damaging
rs4878199	Val253Met	0	Benign

*Adzhubei et al., 2010 [26]

*Abbreviations*: SNP = single nucleotide polymorphism

#### Structural homology modelling

Structural homology models of the monomeric unit of wild type ALDH1B1 (GI: 25777730) were generated using the I-TASSER computational modelling server [27–29]. The top ranked homology model was derived from an alignment with the human ALDH2 protein structure (PDB 1a4zA) (S1 Table). The accuracy of this model was estimated by the I-TASSER server using the topology modelling score [30]. It was subsequently visualized using UCSF Chimera [31]. The model of the wild-type ALDH1B1 was subsequently modified by introducing relevant amino acid substitutions using the rotamer function of UCSF Chimera [31] to allow visualization of the positioning of the three missense variants within the overall structure.

Homology modelling was also used to visualize the structural effects of the stop gain variant rs142427338 (Gln378Ter) and hence the potential damaging effects of this SNP.

#### Molecular dynamic simulations

Molecular dynamics simulations were performed on both the wild-type and the rs2228093 (Ala86Val) variant *ALDH1B1* homology models using GROMACS 5.0 [32, 33]; the rs2228093 variant was selected for study as it is predicted to be the most damaging of the three missense SNPs. The force-field used in all simulations was OPLA-AA/L all-atom force field [34]. The structures were immersed in cubic boxes (size = 10.8 nm3) containing 39842 water molecules. Sodium ions were added for charge neutralization. The Particle Mesh Ewald method [35] was used to treat long range electrostatic interactions and the structures relaxed through steepest descent energy minimization runs until the maximum force was less than 1000 kJ.mol-1.nm-2. The solvent and ions were then equilibrated with the starting structure through two steps of molecular dynamics equilibration runs. The first equilibration was run with a constant number of particles, volume and temperature (NVT) over a period of 100 picoseconds; the presence of a stable temperature profile throughout the run confirmed equilibrium. The second equilibration was run with a constant number of particles, pressure and temperature (NPT) over a period of 100 picoseconds; the presence of stable pressure and density over the run confirmed equilibrium. The final production runs of the molecular dynamics were performed over 100 nanoseconds under NPT conditions.

#### Trajectory analysis

The atomic movements within the output trajectory files from the molecular dynamics simulations were analyzed by root mean square deviation (RMSD) and root mean square fluctuation (RMSF) analyses as performed in GROMACS 5.0 [32,33]. RMSF values were calculated between time points of the simulation at equlibrium indicated by a plateau in the trace of the RMSD trajectories. The RMSD and RMSF values of the variant and wild-type structures were directly compared by visualization of the data points over the entire 100 ns of simulation or the amino-acid sequence of ALDH1B1, respectively. The RMSD and RMSF values were also compared by their average values (mean ±1 standard deviation). Trajectories were animated using visual molecular dynamics (VMD) software [36].

### Genetic association study

#### Alcohol dependent cases

Individuals attending a variety of UK community and hospital-based services providing support and treatment for alcohol use disorders, between 1997 and 2014, were screened for eligibility. They were included in the study if: (i) they met the criteria for alcohol dependence specified in the *Diagnostic and Statistical Manual of Mental Disorders*, *4th Edition* (DSM-IV) [37] or the *International Statistical Classification of Diseases and Related Health Problems*, *10th Revision* (ICD-10) [38]; (ii) if they were of English, Scottish, Welsh or Irish but not Jewish descent with a maximum of one grandparent of European ancestry; and, (iii) they were not related to any of the other included subjects.

#### Controls

Ancestrally-matched controls were recruited from London branches of the National Health Service (NHS) blood transfusion service, from General Practitioners’ surgeries, from amongst university students, and from the general public through the National Institute of Health Research (NIHR) funded Mental Health Research Network (MHRN). Individuals were excluded if they met the schedule for affective disorders and schizophrenia life-time version (SADS-L) criteria for a history of depression, bipolar disorder, schizophrenia or alcohol/drug use disorders [39]; none of the control subjects had a family history of bipolar disorder, schizophrenia or alcohol dependence; none currently drank alcohol above a weekly maximum of 168 g for men or 112 g for women, nor had done so at any time in the past. In addition, DNA from a separate set of controls of British ancestry, not screened for psychiatric or alcohol use disorders, was purchased from the European Collection of Cell Cultures (ECACC; Health Protection Agency Culture Collections, Salisbury, UK).

None of the cases or controls utilized in this study had participated in any previous studies on ALDH genes polymorphisms or in GWAS studies of alcohol dependence.

#### DNA extraction and genotyping

Genomic DNA was extracted from whole blood from using a standard cell lysis, phenol chloroform technique [40]. The ECACC DNA was pre-extracted from transformed lymphoblastoid cell lines.

Primers for genotyping were designed using PrimerPicker software (http://www.lgcgroup.com/genotyping/) (Table 2). Genotyping for the *ALH1B1* SNPs was carried out, in-house, using fluorescent competitive allele specific PCR reagents (KASPar; LGC Genomics, Hoddesdon, UK). Amplification and detection was undertaken using a LightCycler^®^ 480 real time PCR system (Roche Applied Science, Burgess Hill, UK). Genotype calling was performed automatically by built-in Roche software of cluster plots with some manual editing of calls. Approximately 12% of samples, randomly selected *a priori*, were genotyped in duplicate to ensure consistent allele calling.

**Table 2:** Primers used for genotyping the four selected SNPs in *ALDH1B1*.

SNP identity	Sequence of primers
rs2073478	GAAGGTGACCAAGTTCATGCTCCACTAGGTCTGCCAGGC
	GAAGGTCGGAGTCAACGGATTGCTCCACTAGGTCTGCCAGGA
	GGTCCCCATGGCGCCGGAT
	AGCGGGGCCGGCTGCTGAA
rs2228093	GAAGGTGACCAAGTTCATGCTGTGAAAGCAGCCCGGGAAGC
	GAAGGTCGGAGTCAACGGATTCGTGAAAGCAGCCCGGGAAGT
	GAGGCATCCATCCGGCGCCAT
	CATGGGGACCCCAGGCGGAA
rs4878199	GAAGGTGACCAAGTTCATGCTGAAGGCAACTTTGTCAACATCCAC
	GAAGGTCGGAGTCAACGGATTGTGAAGGCAACTTTGTCAACATCCAT
	GTGCGGCCATCGCCCAGCA
	CCCAACAGCAGGTGCGGCCAT
rs142427338	GAAGGTGACCAAGTTCATGCTAGGCTACATCCAGCTTGGCC
	GAAGGTCGGAGTCAACGGATTCTAGGCTACATCCAGCTTGGCT
	GCTCTCCGCCACAGAGGAGTTT
	CAGAGGAGTTTTGCGCCCTCCTT

Abbreviations: SNP = Single nucleotide polymorphism

#### Sequencing of *rs142427338*

Samples identified as carrying the minor allele of the stop gain variant rs142427338 (Gln378Ter), together with samples of uncertain genotype for this SNP, were directly sequenced in order to validate the KASPar genotype calling. Sequencing primers were designed using Primer-BLAST (http://www.ncbi.nlm.nih.gov/tools/primer-blast/) (Forward–CTGACATGGAGCATGCCGT; Reverse–CAGATCCCGGGTGAACACAG). A standard touchdown PCR protocol was performed [41] on 25ng of genomic DNA using a GeneAmp PCR System 9700 machine (Invitrogen, Paisley, UK) with an increased annealing temperature of 68°C to prevent non-specific amplification. PCR amplification success and specificity were determined by visualizing the fragments with agarose gel electrophoresis. Amplified PCR templates were cleaned using PEG precipitation (0.5M NaCl, 1 mM Tris-HCl pH 8.0, 0.1 mM EDTA, PEG 8000 20%, 1.75 mM MgCl2) prior to sequencing. Sequencing reactions were performed using a BigDye terminator kit (Applied Biosystems^®^; Haywards Heath; UK) with an ABI 3730*xl* DNA Analyzer (Applied Biosystems^®^, Life Technologies, California; USA). Sequencing data were read and analyzed using the Staden package [42].

#### Data analysis

Test for primary allelic associations, missingness, deviation from Hardy-Weinberg equilibrium (HWE) and linkage disequilbrium were performed using PLINK version 1.9 [43,44]. Samples with conflicting calls (<0.05% of total) were excluded from further analyses.

### Ethics

United Kingdom National Health Service Multicentre Research Ethics Committee approval was granted for this study (MREC/03/11/090). This was ratified by the Research and Development Departments associated with the individual participating centres. Written informed consent was obtained from all subjects prior to inclusion.

## Results

### Bioinformatic analysis

#### Predicted Structure of *ALDH1B1*

The top ranked predicted structure for the ALDH1B1 monomeric unit had a 95% structural overlap and 76% amino acid sequence identity with the ALDH2 structure 1A4Z. The estimated template modelling score between the homology model structure and the undetermined native structure was 0.69 ± 0.12.

**Fig 1:**
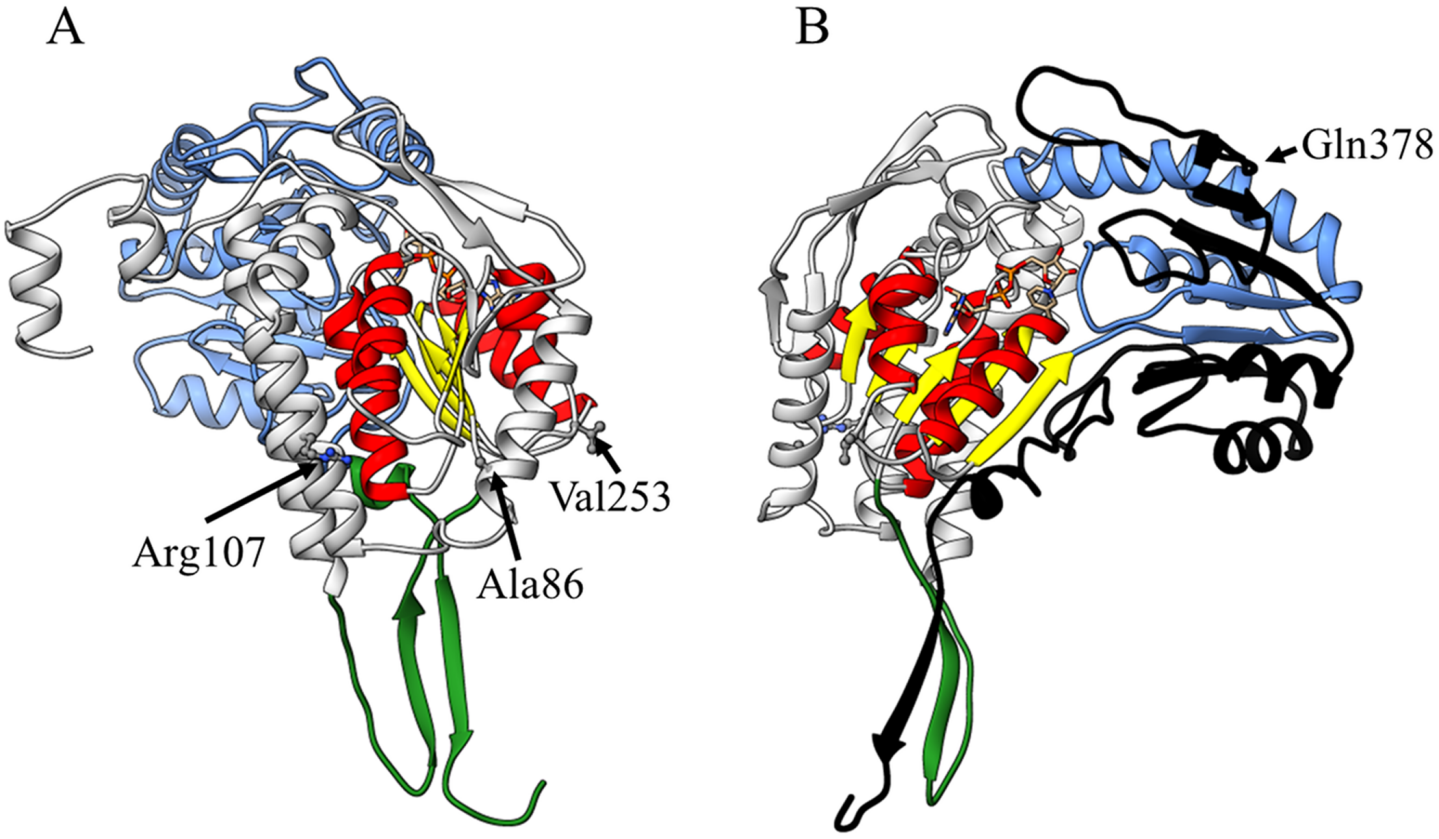
Homology model structure of the wild-type ALDH1B1 primary sequence (GI: 25777730). This model structure is annotated and rotated to illustrate the positionings of: A) the three misssense variants; and, B) the stop codon variant.

The key structural features of the predicted ALDH1B1 monomer subunit were: (i) a dinucleotide binding domain; (ii) an oligomerization domain; and, (iii) a catalytic domain (Fig 1A; S1 Video). The three missense variants rs2073478 (Arg107Leu), rs2228093 (Ala86Val) and rs4878199 (Val253Met) are predicted to locate to the co-enzyme domain facing the outer surface of the enzyme (Fig 1A). The nonsense variant rs142427338 (Gln378Ter) is predicted to result in a premature transcription termination codon within *ALDH1B1* and hence loss of 139 amino acids in the catalytic domain of the enzyme crucial for its function (Fig 1B).

#### Molecular dynamics simulations

**Fig 2:**
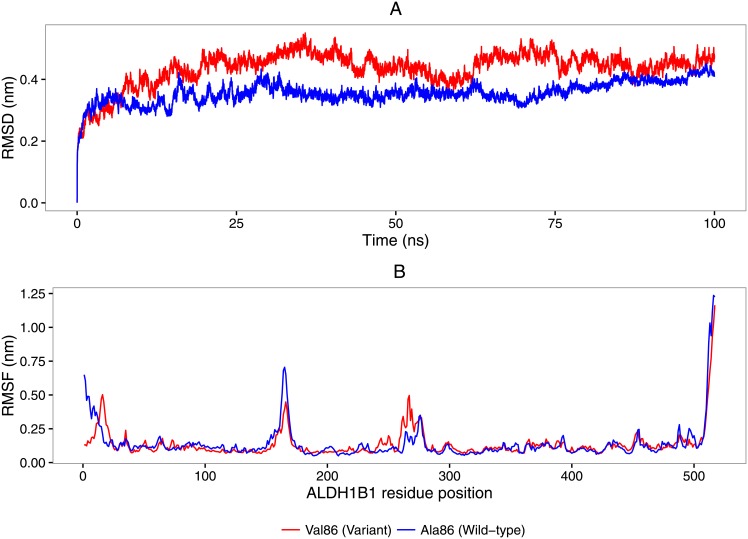
Molecular dynamics simulations performed on the structural homology models of the wild type and the 86Val variant structure of ALDH1B1. A—The RMSD* of carbon α backbone atoms over the course of the entire 100 ns molecular dynamics simulation for the wild-type (blue) and Val86 variant (red) structure. B—The RMSF** of carbon α backbone atoms over the last 90 ns of the molecular dynamics simulation for the wild-type (blue) and Val86 variant (red) structure. * The root mean squared deviation (RMSD) is a measure the average distance between a number of atomistic positions between a reference structural model and the structural model at a point in time during the simulation. Its variability over the time-course of the simulation indicates the scale of atomistic movement in a structural model and whether it has achieved dynamic equilibrium. ** The root mean squared fluctuation (RMSF) is a calculation of the average RMSD in a well-defined atomistic position, typically atoms present in key regions of the protein structure, over a specified time-period of the simulation. The RMSF highlights differences in movement between in a protein structure over a time period of a simulation and is useful for comparing two or more simulation trajectories.

The RMSD of the carbon αbackbone of the wild-type ALDH1B1 monomer indicates that the structures attained equilibrium deviation after 10 ns (Fig 2A). The RMSF indicates that this monomer contains four major regions of structural flexibility *viz*: (i) a region around residues 1–17 in the N-terminal, which lies within the mitochondrial targeting sequence of the enzyme; (ii & iii) two regions around residues 150–170 and 510–517 which, despite being distant in the primary sequence, are sterically close within the monomer structure and encompass the majority of the oligomerization domain; and, (iv) a region around residues 270–280 which contains an alpha-helix α*D*, which is a key component of the dinucleotide binding domain within the Rossman fold (Fig 2B; S1 Video).

The simulation trajectory of the Val86 variant monomer differed from that of the wild type (average RMSD ± SD = 0.44 ± 0.055 nm *vs*. average RMSD ± SD = 0.35 ± 0.034 nm respectively), suggesting that the Val86 substitution reduces the structural stability *in silico*. The RMSF data generally mirrored those of the wild-type, except for: (i) a reduction in the flexibility between residues in positions 150–170, which correspond to a region of the oligomerization domain; and, (ii) an increase of the flexibility between residues in positions 270–280, which correspond to a key component of the dinucleotide binding domain.

### Genetic association analysis

#### Genotyping accuracy

Genotyping was completed for all four SNPs with call rates all greater than 95%. All markers followed Hardy Weinberg Equilibrium (HWE; cut-off > 0.05) in cases and controls.

#### Primary allelic associations of *ALDH1B1* SNPs

A total of 1478 alcohol dependent cases and 1254 controls were genotyped.

No significant differences were observed in the frequencies of the three missense *ALDH1B1* SNPs between the cases and controls (Table 3). However, the prevalence of the allele of the rare rs142427338 (Gln378Ter) was higher in the cases (allelic = 0.19, odds ratio [OR] = 2.98, 95% confidence interval [CI] 0.62–14.37) (Table 3). The variants were in strong linkage disequlibrium.

**Table 3:** Single marker allelic associations for genotyped *ALDH1B1* polymorphisms in alcohol dependent cases and controls.

SNP	Cohort	Number	Minor allele	Genotype counts			MAF (%)	Significance (*P* value)*	OR (95% CI)
rs2073478				**TT**	**TG**	**GG**			
	Case	1446	**G**	508	698	240	40.7	0.75 (1)	1.03 (0.88–1.20)
	Control	1244		433	604	207	40.9		
rs2228093				**CC**	**CT**	**TT**			
	Case	1450	**T**	1080	346	24	13.6	0.91 (1)	0.99 (0.89–1.11)
	Control	1245		939	282	24	13.3		
rs4878199				**AA**	**AG**	**GG**			
	Case	1415	**G**	1299	114	2	4.2	0.42 (1)	0.90 (0.69–1.17)
	Control	1232		1122	106	4	4.6		
rs142427338				**CC**	**CT**	**TT**			
	Case	1472	**T**	1466	6	0	0.2	0.19 (0.77)	2.98 (0.62–14.37)
	Control	1252		1250	2	0	0.1		

*Abbreviations*: SNP = Single Nucleotide Polymorphism; MAF = Minor Allele Frequency; χ^2^ = Chi-squared statistic; OR = Odds Ratio; CI = Confidence Intervals.

*The unadjusted significance value of an allelic Fishers exact test; the value in brackets has been adjusted for multiple testing using Bonferroni correction.

## Discussion

The missense variant rs671 in *ALDH2* significantly affects the risk of developing alcohol dependence and its sequelae in East Asian populations but this variant is largely absent in European populations. There is, however, significant sequence homology between *ALDH2* and *ALDH1B1* and similarities in their enzymes cellular positioning within the mitochondrion and in their tendency to forms homo-tetramers *in vitro*. Importantly, however, *ALDH1B1*, unlike *ALDH2*, contains several missense SNPs which are common in European populations. Nevertheless, it is unclear whether these variants affect the processing of acetaldehyde, and hence influence the risk of developing the adverse consequences associated with alcohol consumption. This was addressed in the present study, in which four non-synonymous variants in *ALDH1B1*, three missense and one stop gain, which result in changes to the primary sequence of ALDH1B1, were further characterized using functional prediction software, structural homology modelling and molecular dynamics simulation.

The structure of the ALDH1B1 monomer subunit was predicted using the top-ranked I-TASSER software [28]. The output metrics for the final homology model provided a high degree of confidence in the prediction [30]. The technique of molecular dynamics simulation was used to infer the flexibility of this predicted ALDH1B1 structure and characterize the effects of the 86Val allelic variant on protein structure and function [45,46]. The simulations revealed four regions of flexibility. The first, located at the N-terminal of the protein (residues 1–17), encompassed the mitochondrial targeting sequence. The simulated flexibility in this mitochondrial targeting sequence agrees with Nuclear Magnetic Resonance (NMR) and crystallography studies, which have experimentally determined freedom of movement in this region in homologous ALDH proteins [47,48]. The second and third regions of flexibility (residues 150–170 and 510–517) lie in the oligomerization domain of ALDH1B1. Flexibility in this domain might reflect the fact that the simulation left this region exposed whereas in the putative ALDH1B1 tetramer the oligomerization domain would likely be buried and hence stabilized within the overall macromolecular structure. The fourth region of flexibility (residues 270–280) contains an alpha-helix α*D* which is a key component of the dinucleotide binding domain. Structural studies of the homologous alpha-helix in ALDH2 comparing the *apo* and *holo* forms of the enzyme, have shown that movement of this alpha-helix is essential for isomerization of bound NAD, a significant and necessary feature of catalysis [49].

A comparison of the molecular dynamics trajectory of the predicted structures of the wild-type ALDH1B1 and the missense variant predicted to be most damaging *viz*. rs2228093 (Ala86Val) demonstrates a considerable increase in the flexibility of the 270 to 280 region. As this region of the protein is crucial for the binding of the coenzyme NAD, this observation could explain the catalytic inactivity of this variant previously observed *in vitro* [19]. Ultimately, however, this technique does not provide conclusive evidence of a functional effect of this genetic variant on protein structure but does provide a basis for later confirmation by X-ray crystallography or solution NMR. This, not withstanding, other studies have reported [50] excellent correlations between molecular dynamics simulation and experimental data.

The variant, rs142427338 (Gln378Ter) in *ALDH1B1* encodes a stop codon rather than another amino-acid residue. Such ‘loss of function’ or ‘nonsense’ variants may exert functional effects at either the transcript level, *via* haploinsufficiency mechanisms, or at the protein level, *via* truncation mechanisms. Haploinsufficiency occurs when the mRNA transcript encoding a nonsense allele is eliminated *via* nonsense mediated decay (NMD). The process of NMD, however, requires splice sites for the detection of premature stop codons and thus does not occur on transcripts of intronless genes [51]; as *ALDH1B1* is intronless NMD seems unlikely. Thus, if this transcript is translated and the protein product does not invoke the unfolded protein response, the loss of function allele of *ALDH1B1* could result in loss of a sizeable portion of the final enzyme product encompassing the C-terminal 139 amino acids. This region of the protein contains the enzyme’s catalytic residues and may also be important for tetramer formation. Hence, an ALDH1B1 protein truncated at position 378 would likely be non-functional. Clearly experimental studies would greatly facilitate functional understanding of this variant.

Despite the strength of evidence, from the bioinformatic analyses, for the presence of potentially damaging functional mutations in *ALDH1B1*, these coding variants were not significantly associated with alcohol dependence *per se* in a large well-characterized population of alcohol dependent cases and controls of British and Irish ancestry. However, the prevalence of the 378STOP allele in rs142427338 (Gln378Ter) was higher in cases than in controls although the difference was not significance. The MAFs of the three missense variants ranged from 4.6% to 40.9% and hence this study was sufficiently powered to detect significance between cases and controls. However, the stop gain variant is very rare with a MAF of 0.01; thus this study was likely underpowered to detect genetic association at the current sample size [52].

There are very few genetic association data available for comparison. Sherman et al. [20], found no association between carriage of either rs2073478 or rs2228093 and alcohol dependence risk in a cohort of 40 people of British ancestry genotyped using PCR-RFLP. Unfortunately, the size of this population severely limits the statistical power at the expected effect size [53]. Additionally, the MAFs reported by Sherman et al [20] were notably different from those in the present study *viz* 29% and 41% respectively for rs2073478 and 24% and 13.3% respectively for rs2228093; this may reflect inaccuracies in the calculation of the population MAF due to the small size of the cohort and/or to inaccuracies arising from use of PCR-RFLP genotyping, which is prone to partial digestion and genotype reading errors [54]. However, the present study is sufficiently powered to allow confidence in the concordant, negative association [52].

The relationships between polymorphisms in *ALDH1B1* and alcohol drinking levels in the general population and alcohol-related hypersensitivity reactions have been examined in two large populations of Danish residents of Northern European origin [23–25]. The reported MAFs for the two SNPs examined were similar to those in the present study *viz* 39% and 41% respectively for rs2073478 and 12% and 13% respectively for rs2228093; thus the studies can be usefully compared.

In the first Danish study Husemoen *et al*. [23] obtained self-reported information on alcohol consumption from 1216 participants in the population-based Copenhagen Allergy Study and reported that carriage of the minor allele of rs2228093 was associated with non-drinking and *higher* weekly alcohol intake; these findings are paradoxical as the same directionality of allele effect between these related phenotypes would be expected.

In the second study, Linneberg *et al*. [25] combined the information on alcohol consumption obtained from their original cohort of 1216 with similarly collected information from an additional 6784 participants in a replication study. In addition they obtained self-reported information on alcohol hypersensitivity and allergic rhinitis from 957 (79%) of the original 1216 participants and 2419 (36%) of the 6784 replication cohort. In the combined population, carriers of the minor allele of rs2228093 reported significantly *higher* total alcohol consumption and an *increased* prevalence of alcohol hypersensitivity; this finding is again paradoxical as one would expect alcohol hypersensitivity to be associated with lower total alcohol consumption and thus expect a common risk allele between phenotypes. It is unclear why this apparent paradoxical effect occurs, although it is possible that the reported allergic reactions may have been too mild to warrant avoidance of alcohol. However, quantitative information on the severity of the response was not made available.

Emerging evidence suggests that rs2228093 has been under positive selection in East-Asian populations over the past 10,000 years [55]. Wang *et al*. [55] showed that several functional SNPs in alcohol metabolizing genes *viz* rs1229984 (*ADH1B*), rs671 (*ALDH2*), rs8187929 (*ALDH1A1*), rs3813867 (*CYP2E1*) and rs2031920 (*CYP2E1*), have been under moderate to strong selection, which appears to have been driven by the expansion of agriculture. Although these findings are not directly related to alcohol use phenotypes *per se*, they highlight the inter-related function of alcohol metabolizing genes and in particular the shared factors influencing allele frequencies in these populations. If selection pressures aresimilarly influencing these genes in Europeans, then it is possible that the resultant effects could confound genetic association studies of *ALDH1B1*, potentially explaining the negative findings observed in the present study and the paradoxical findings observed by the Danish groups [23,25]

## Conclusion

In conclusion: bioinformatic techniques have been used to investigate missense SNPs in *ALDH1B1*, in particular the 86Val allele of rs2228093, which is predicted to disrupt the structural flexibility of the protein product, the enzyme ALDH1B1. If, as indicated from the bioinformatic data, these changes influence NAD binding to the catalytic domain of the enzyme, or oligomerization between the enzyme subunits, then they could alter enzyme activity. Functional and molecular cloning studies would be needed to further elucidate the effects of these polymorphisms and, in particular, their physiological role in alcohol pharmacokinetics. Although no genetic associations were identified between the four selected SNPs in *ALDH1B1* and alcohol dependence, the study was likely underpowered in relation to the stop gain variant rs142427338 due to its rarity in this population. Exploration of these *ALDH1B1* variants within other European populations is clearly warranted.

## Supporting information

S1 Fig *ALDHB1* variant allele frequencies in four European ancestry 1000 genomes sub-populations. Major and minor allele frequencies for the *ALDH1B1* variants rs2073478, rs2228093 and rs4878199 are shown. The four European ancestry sub-populations include: (A) Finnish in Finland (FIN); (B) British in England and Scotland (GBR); (C) Iberian Population in Spain (IBS); and, (D) Toscani in Italia (TSI).
(TIFF)

S1 Table The top ten templates used by to generate the ALDH1B1 structural homology model from the wild-type ALDH1B1 primary sequence (GI: 25777730). *PDB hit*: The protein databank ID of the template structure used to generate the ALDH1B1 homology model. *Identity1*: The percentage sequence identity of the template structure and the ALDH1B1 primary sequence. *Identity2*: The percentage sequence identity of the ALDH1B1 primary sequence and the template structure; *Coverage*: The percentage of residues which align between the template structure and the ALDH1B1 primary sequence. *Normalized Z-score*: A metric of template alignment accuracy: scores > 1 indicate good alignment.
(DOC)

S1 Video An overview of the ALDH1B1 structural model and molecular dynamics simulation. A short video summarizing the key-features of the ALDH1B1 structural model and molecular dynamics simulation.
(ZIP)

S1 File Sequencing and genotype call data. The double-anonymised genotype calling data and sequencing data used in the present study. This supplemental file directory contains two sub-directories: 1. "Sequencing", which contains the Sanger sequencing data (trace and sequence call data) for those samples that underwent sequencing verification for the variant rs142427338. 2. "Genotypes", which contains PLINK compatible data (.ped and.map) containing the final genotype calls for the four ALDH1B1 variants analysed.
(ZIP)
