# Supplementary figures and images for: Genetic variants in ALDH1B1 and alcohol dependence risk in a British and Irish population: A bioinformatic and genetic study
Source: PLoS One. 2017 Jun 8;12(6):e0177009. doi: 10.1371/journal.pone.0177009 (PMC5464525; doi:10.1371/journal.pone.0177009)

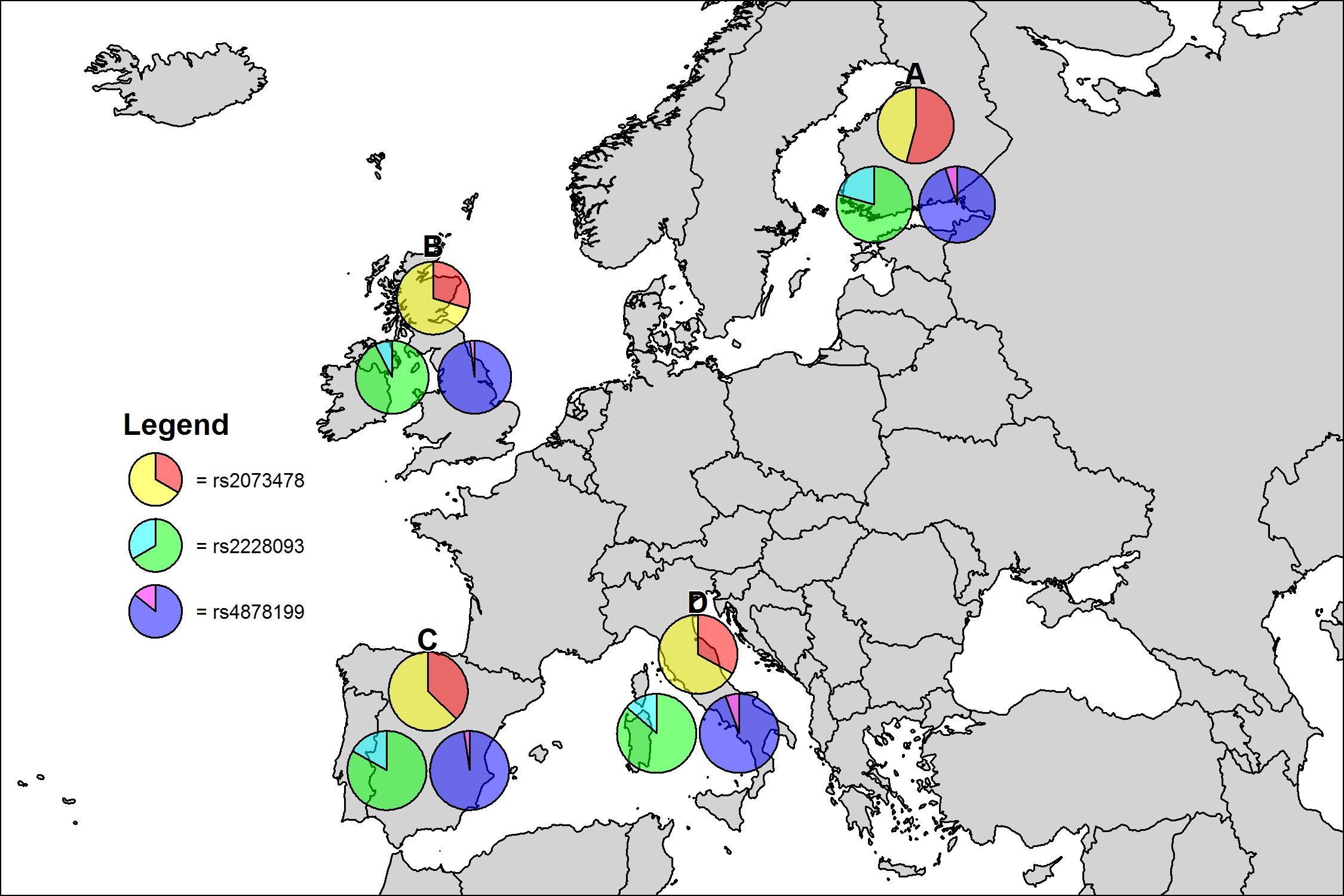

Supplement: S1 Fig — Major and minor allele frequencies for the ALDH1B1 variants rs2073478, rs2228093 and rs4878199 are shown. The four European ancestry sub-populations include: (A) Finnish in Finland (FIN); (B) British in England and Scotland (GBR); (C) Iberian Population in Spain (IBS); and, (D) Toscani in Italia (TSI). (TIFF) [file pone.0177009.s001.tiff]
