# Supplementary material for: Genetic variants in ALDH1B1 and alcohol dependence risk in a British and Irish population: A bioinformatic and genetic study
Source: PLoS One. 2017 Jun 8;12(6):e0177009. doi: 10.1371/journal.pone.0177009 (PMC5464525; doi:10.1371/journal.pone.0177009)
Supplement: S1 Table — PDB hit: The protein databank ID of the template structure used to generate the ALDH1B1 homology model. Identity1: The percentage sequence identity of the template structure and the ALDH1B1 primary sequence. Identity2: The percentage sequence identity of the ALDH1B1 primary sequence and the template structure; Coverage: The percentage of residues which align between the template structure and the ALDH1B1 primary sequence. Normalized Z-score: A metric of template alignment accuracy: scores > 1 indicate good alignment. (DOC) [file pone.0177009.s002.doc]

**S1 Table. The top ten templates used by to generate the ALDH1B1 structural homology model from the wild-type ALDH1B1 primary sequence (GI: 25777730).**

| **Rank** | **PDB hit** | **Identity1** | **Identity2** | **Coverage** | **Normalized Z-score**  **(0-8)** |
| --- | --- | --- | --- | --- | --- |
|  |  | **(%)** | | |  |
| 1 | 1a4zA | 76 | 72 | 95 | 4.20 |
| 2 | 2wmeA | 41 | 39 | 93 | 2.24 |
| 3 | 4a0mA | 41 | 40 | 93 | 4.11 |
| 4 | 1bxsA | 65 | 62 | 96 | 5.97 |
| 5 | 1a4zA | 76 | 72 | 95 | 3.88 |
| 6 | 1nzwA | 75 | 71 | 96 | 7.10 |
| 7 | 2wmeA | 41 | 39 | 93 | 0.26 |
| 8 | 4a0mA | 41 | 40 | 93 | 4.01 |
| 9 | 1a4zA | 76 | 72 | 95 | 3.24 |
| 10 | 1bxsA | 65 | 62 | 96 | 4.12 |

***PDB hit:*** The protein databank ID of the template structure used to generate the ALDH1B1 homology model

***Identity1:*** The percentage sequence identity of the template structure and the ALDH1B1 primary sequence

***Identity2:*** The percentage sequence identity of the ALDH1B1 primary sequence and the template structure;

***Coverage:*** The percentage of residues which align between the template structure and the ALDH1B1 primary sequence

***Normalized Z-score:*** A metric of template alignment accuracy: scores > 1 indicate good alignment
